# State of the Art in Carbon Nanomaterials for Photoacoustic Imaging

**DOI:** 10.3390/biomedicines10061374

**Published:** 2022-06-10

**Authors:** Moon Sung Kang, Haeni Lee, Seung Jo Jeong, Tae Joong Eom, Jeesu Kim, Dong-Wook Han

**Affiliations:** 1 Department of Cogno-Mechatronics Engineering, College of Nanoscience & Nanotechnology, Pusan National University, Busan 46241, Korea; mskang7909@gmail.com (M.S.K.); haenilee@pusan.ac.kr (H.L.); 2 Bio-IT Fusion Technology Research Institute, Pusan National University, Busan 46241, Korea; biomechanics@nate.com

**Keywords:** carbon nanomaterials, phototherapy, photoacoustic imaging, image-guided therapy

## Abstract

Photoacoustic imaging using energy conversion from light to ultrasound waves has been developed as a powerful tool to investigate in vivo phenomena due to their complex characteristics. In photoacoustic imaging, endogenous chromophores such as oxygenated hemoglobin, deoxygenated hemoglobin, melanin, and lipid provide useful biomedical information at the molecular level. However, these intrinsic absorbers show strong absorbance only in visible or infrared optical windows and have limited light transmission, making them difficult to apply for clinical translation. Therefore, the development of novel exogenous contrast agents capable of increasing imaging depth while ensuring strong light absorption is required. We report here the application of carbon nanomaterials that exhibit unique physical, mechanical, and electrochemical properties as imaging probes in photoacoustic imaging. Classified into specific structures, carbon nanomaterials are synthesized with different substances according to the imaging purposes to modulate the absorption spectra and highly enhance photoacoustic signals. In addition, functional drugs can be loaded into the carbon nanomaterials composite, and effective in vivo monitoring and photothermal therapy can be performed with cell-specific targeting. Diverse applied cases suggest the high potential of carbon nanomaterial-based photoacoustic imaging in in vivo monitoring for clinical research.

## 1. Introduction: Carbon Nanomaterial for Photoacoustic Imaging

**Figure 1:**
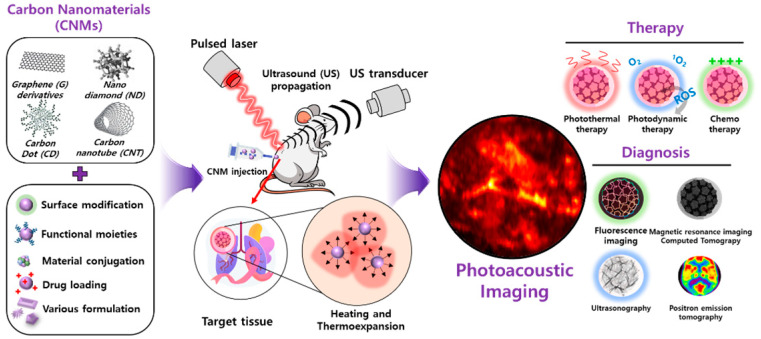
Illustration of functionalized carbon nanomaterial for photoacoustic imaging and a multimodal theragnostic probe.

The discovery of carbon nanomaterials (CNMs) has impacted many aspects of nanotechnology and has contributed to significant developments in physics, electronics, optics, mechanics, biology, and medicine [1,2,3]. CNMs exhibit fast electron transfer kinetics and better electrocatalytic activity, unique magnetic and optical properties, chemical versatility with ease of manipulation, biocompatibility, and performance as a chemically robust platform [4,5]. Herein, we evaluate the potential of CNMs for their photoacoustic imaging (PAI) application by categorizing them into graphene (G) and their derivatives (i.e., graphene oxide (GO) and reduced graphene oxide (rGO), carbon nanotubes (CNTs), nanodiamonds (NDs), carbon dots (CDs), and diverse types of carbon nanoparticles (CNPs)). The formulation of CNMs, including surface modification, functionalization with drugs/inorganic/organic materials, and fabrication method, enhances both PA signal intensity and theragnosis versatilities (Figure 1).

## 2. Photoacoustic Imaging

In biomedical studies, various imaging techniques such as magnetic resonance imaging (MRI) [6], positron emission tomography (PET), X-ray computed tomography (X-ray), ultrasound imaging (USI) [7,8], and optical imaging techniques have been used for monitoring biological response in small animals. Among the imaging techniques, photoacoustic imaging (PAI) has widely been explored in the last two decades since it has unique characteristics through inherited complementary advantages from optical imaging and USI [9]. Similar to pure optical imaging techniques, PAI can provide molecular information from the multispectral photoacoustic (PA) responses of biological tissues [10]; thus, it can be used for functional imaging, such as monitoring hemoglobin oxygen saturation levels (sO_2_) [11], investigating melanin components [12,13,14], and detecting lipids [15]. However, unlike optical imaging methods, the photon diffusion in biological tissues usually does not affect the quality of PA images because the signal reception part of PAI is inherited from USI, which has a relatively high resolution in deep tissue. Therefore, PAI can be applied to intermediate applications of optical imaging and USI, such as visualizing the molecular information of biological tissue a few centimeters deep.

**Figure 2:**
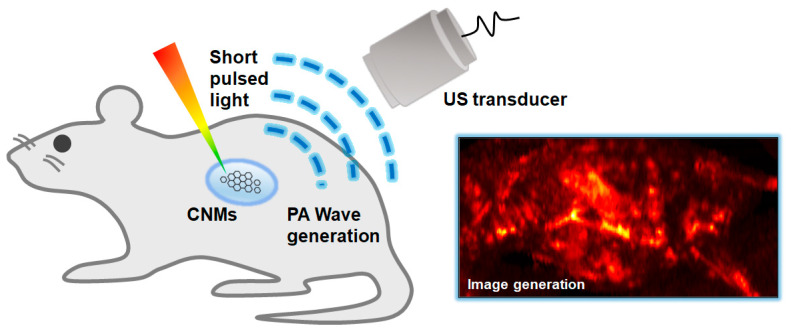
Schematic diagram of the principles of the PA imaging technique. PA, photoacoustic; US, ultrasound; CNMs, carbon nanomaterials.

The underlying principle of PAI is the PA effect [16], which is an energy transformation from light to acoustic wave (Figure 2). In PAI, a pulsed laser is delivered to the target objects. The optically absorbing chromophores in biological tissues absorb the delivered light energy and convert it to heat energy, which is quickly dissipated since the pulse width of the laser is very short (typically a few nanoseconds) [17]. This quick heat change generates acoustic waves called PA waves through thermoelastic expansion. The vibration propagates as a form of acoustic wave called PA waves. The propagated PA waves are then detected by conventional ultrasound (US) transducers and then converted to digital signals for image generation. By using the conventional image generation process in USI, PA images can also be reconstructed. Therefore, in PAI, the optical absorption characteristics of biological tissues are visualized through acoustic propagation, providing molecular functional information with relatively high US resolution in deep tissue, because the scattering of acoustic waves is much less than that of optical photons. PAI can significantly enhance the imaging depth (up to a few centimeters) compared to pure optical imaging techniques (typically limited to 1 mm).

From the principles of PAI, we can note that signal generation is determined by the amount of energy transformed from light to acoustic waves. The initial pressure P [Pa] of the PA wave is related to the following four parameters: (1) the Grüneisen coefficient Γ (T), which is a function of the local temperature T, (2) the optical absorption coefficient $\mu_{a}$ (cm^−1^) that is determined by the concentration of chromophores and the wavelength of the excitation laser, (3) the heat conversion efficiency 0 ≤ σ ≤ 1 (dimensionless), and (4) the optical fluence F (J∙cm^−2^). Interestingly, those four parameters are linearly proportional to the initial pressure, as given in the following equation; thus, the PA signal can be easily amplified by increasing one of the four parameters.

(1) $$P\propto \Gamma \left(T\right)\cdot \mu_{a}\cdot \sigma \cdot F.$$

Most importantly, when the excitation wavelength varies, the optical absorption coefficient *μ_a_* varies accordingly. Consequently, the spectral PA responses enable us to assess the composition of biological tissues based on the unique optical absorption spectra of chromophores.

The biological chromophores, such as oxyhemoglobin, deoxyhemoglobin, melanin, and lipid, can be used as intrinsic contrasts in PAI. Although the intrinsic chromophores are visible in PAI, various exogenous agents have also been widely utilized for contrast-enhanced imaging for visualizing optically transparent objects such as the lymphatic system [18], bladder [19,20], and tumors [21,22]. In addition to the contrast-enhancing in PAI, recent agents have also been functionalized for light-activated therapy [23,24,25]. By monitoring the therapeutic responses in vivo, the efficacy of developed drugs can be assessed.

In this paper, we summarize and review the recent carbon nanomaterials for monitoring drug delivery and therapy monitoring using PAI. We first explore the types of configurations of PAI systems for the in vivo visualization of biological systems. Then, the representative PAI results are reviewed. The results show the great potential of PAI for various biomedical studies using carbon nanomaterials.

## 3. System Configurations for Photoacoustic Imaging

One unique characteristic of PAI is that the resolution and imaging depth can be scalable according to the configuration of the system. By selecting appropriate systems, various scales of biological targets, from organelles to organs, can be imaged [26]. The two major branches of PAI configuration are PA microscopy (PAM) and PA computed tomography (PACT); PAM is further divided into optical-resolution PAM (OR-PAM) and acoustic-resolution PAM (AR-PAM). In this section, the typical implementation and performance of each type of PAI system are benchmarked (Table 1).

## 4. Photoacoustic Microscopy

**Figure 3:**
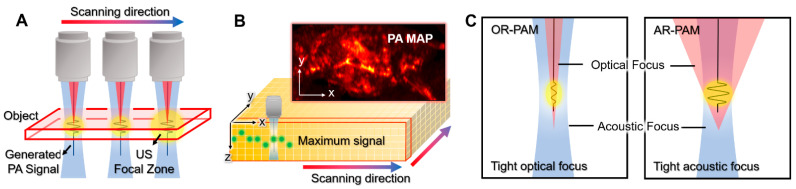
Schematic illustrations of PAM. (**A**) Scanning mechanism of PAM. (**B**) PA MAP image generation from volumetric data. (**C**) The optical and acoustic foci in OR-PAM and AR-PAM. PA, photoacoustic; PAM, photoacoustic microscopy; US, ultrasound; MAP, maximum amplitude projection; OR-PAM, optical-resolution PAM; AR-PAM, acoustic-resolution PAM.

PAM systems typically use a single-element US transducer with a fixed focal zone to achieve high resolution (Figure 3A). Because of the fixed focal region, the achieved data can be considered from an axial line of the transducer. Therefore, PAM does not require complex mathematical computation for image reconstruction. In addition, since the speed of sound in biological tissue is relatively slow (1540 m/s in soft tissue), the PA waves captured by the US transducer are congenitally depth-resolved signals along the depth direction (i.e., A-line signals). By scanning the transducer or the optical path in the transversal plane, volumetric data in the scanning area can be acquired (Figure 3B). To visualize the volumetric information in a plane, the volumetric data are projected on the transversal plane using the maximum amplitude projection (MAP) method.

The key difference between OR-PAM and AR-PAM is the focusing of light, which determines the scale of resolution. In OR-PAM, the focal region of the optical beam is smaller (typically 10-fold) than that of the acoustic focal region (Figure 3C); thus, the lateral resolution of OR-PAM ($r_{O}$) is determined by the size of the optical focal zone [39,40,41,42]. The lateral resolution of OR-PAM can be expressed as follows [43].

(2) $$r_{O}=0.51\cdot \frac{\lambda_{O}}{NA_{O}}$$

where $NA_{O}$ is the optical numerical aperture, and $\lambda_{O}$ is the wavelength of the excitation light. The tight optical focus gives OR-PAM a very high resolution (sub-µm to 5 µm), but the imaging depth is limited in the optical ballistic regime (<1 mm) because the optical focus cannot be maintained beyond the optical mean free path. Therefore, the typical applications of OR-PAM are organelles in the superficial region, such as mitochondria [26] and red blood cells [44], in small animals.

On the other hand, the optical beam in AR-PAM is widely diffused on the target sample; thus, the resolution of AR-PAM is determined by the acoustic focal size (Figure 3C). The lateral resolution of AR-PAM (*r_A_*) depends on the acoustic properties, as follows [43].

(3) $$r_{A}=0.71\cdot \frac{\lambda_{A}}{NA_{A}}=0.71\cdot \frac{v_{s}}{NA_{A}\cdot f_{c}}$$

where $NA_{A}$ is the numerical aperture of the US transducer, $\lambda_{A}$ is the center wavelength of the detected PA wave, $v_{s}$ is the speed of sound (~1540 m/s in soft tissue), and $f_{c}$ is the center frequency of the US transducer. Although the lateral resolution is relatively low compared to OR-PAM, AR-PAM has the benefit of extending the imaging depth to several millimeters since a tight optical focus is not required. Therefore, AR-PAM typically operates in the optical diffusive regime (>1 mm). In addition, the field-of-view (FOV) in AR-PAM can be increased to the whole-body of mice; thus, the applications are expanded to monitoring the whole-body distribution of agents [45], the identification of sentinel lymph nodes (SLN) [46], and the visualization of tumors [47].

## 5. Photoacoustic Computed Tomography

The main purpose of PACT is fast image acquisition in a larger area, which includes a wider imaging area in the transversal plane and a deeper imaging depth compared to PAM. Unlike PAM, PACT uses various geometries (e.g., linear, curved, and spherical) of array transducers. Therefore, PACT requires mathematical computations to generate an image. The image reconstruction algorithms also generate artifacts in the resulting images; hence, the image quality is relatively low compared to the images in PAM. However, because PACT can visualize tomographic information in biological tissue with fast imaging speed, it has been widely applied to various biomedical studies that require a series of deep tissue monitoring.

**Figure 4:**
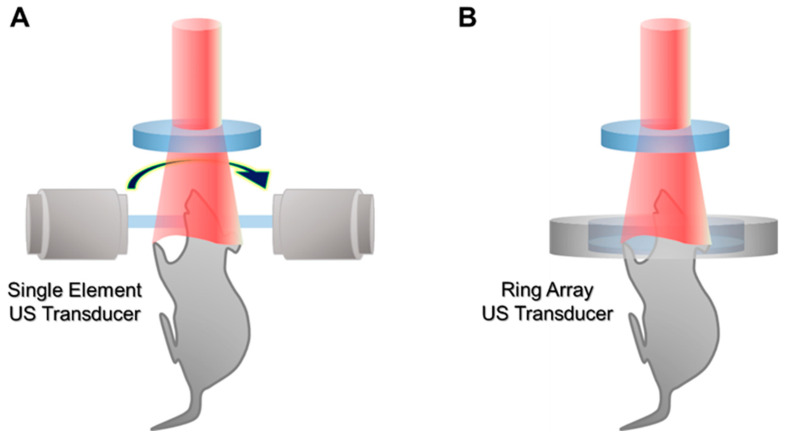
Schematic illustration of PACT. (**A**) PACT with a rotational scanning of a single-element US transducer. (**B**) PACT with a ring array transducer. PACT, photoacoustic computed tomography; US, ultrasound.

In the initial stage, PACT uses the rotational scanning of a single-element transducer to achieve PA waves in multiple directions (Figure 4A). The initial systems showed promising results for visualizing tomographic images of mice, especially the brain [48,49]. However, the traditional rotational scan suffers from slow imaging speed and a relatively complex system configuration. Recently, array transducers in the shape of rings or arcs have been investigated to enhance imaging speed (Figure 4B) [50,51,52]. Since the array transducers can achieve PA signals in multiple directions at once, the imaging speed of PACT can be greatly increased; even real-time imaging is possible [53]. In addition to the ring array transducer, PACT adopts various types of transducers, such as linear [54], hemispherical [55], planar [56], and bowl [57] transducers. The array transducers enable PACT to acquire 2D or 3D images with much faster imaging speeds compared to traditional systems with the scanning of single-element transducers.

More recently, PACT has been implemented in clinical USI machines for the potential clinical translation of the PAI technique [58,59,60]. From the principles, the signal reception and image generation of PAI and USI can be shared on a single platform. In addition to the similarity of image generation, dual-modal PA and USI (PAUSI) can provide complementary information that may be useful in clinical applications. In the overlaid images, both the anatomical (from USI) and metabolomic (from PAI) information of biological tissue can be simultaneously visualized. Therefore, the combined PAUSI information has been applied to various clinical human studies, including thyroid cancer detection [9,61,62], prostate cancer imaging [63,64], skin disease assessment [13,14], inflammation detection [65], and peripheral vasculature imaging [66,67].

## 6. Graphene-Based Photoacoustic Imaging

G is one of the most highlighted two-dimensional (2D) nanomaterials; it is composed of a single layer of an sp2-carbon network. Owing to its exceptional physical, optical, electrical, and biological properties, recent studies have emphasized the biomedical potential of G, including tissue engineering scaffolds, medical devices, in vivo or in vitro bioimaging probes, drug delivery systems, and biosensors. G-derivatives, including GO and rGO, are known to possess extraordinary characteristics. GO is an oxidized form of G that is functionalized with hydrophilic moieties, including hydroxyl, epoxy, carbonyl, and carboxylic groups. On the other hand, rGO is a reduced form of GO that features functional defects in its basal planes; this enables various loading of other materials, drugs, proteins, and imaging dyes while maintaining some functional group residues of GO. Owing to their characteristic physicochemical properties, the development of novel PAI probes has adopted GO and rGO to enhance PA signal intensity and for multimodal application.

Indocyanine green (ICG) is a Federal Drug Administration (FDA)-approved agent for clinical imaging; it exhibits strong NIR absorption and FL emission [68,69]. However, ICG has limited clinical application due to concentration-dependent aggregation, low photostability, aggregation with proteins, and a short half-life in circulation [70]. To overcome these issues, GO–ICG has been developed by Wang et al. by functionalizing ICG and modified FA onto GO nanocomposites, which allow PTT and PAI multimodal application [71]. The ICG–GO–FA composites showed a 10-fold enhanced PA signal at cell imaging compared to PBS. Notably, FA modification allowed the active targeting of folate receptor-expressing tumor cells, while there was no significant cytotoxicity without NIR irradiation to normal cells. The folate receptor is a glycosyl-phosphatidylinositol conjugated glycoprotein that is overexpressed on cancer cell membrane surfaces [72]. The FA-conjugated probes can be endocytosed via the folate receptor; hence, specific cancer targeting can be achieved [8,73].

The ICG–GO–FA showed dose-dependent cytotoxicity to HeLa cells, demonstrating that 20 μg/mL of treatment is not significantly cytotoxic. When 808 nm laser irradiation was used, ICG–GO–FA-treated HeLa cells were necrotized, but there was no toxicity to normal cells, suggesting ICG–GO–FA is a promising agent for the targeted PT ablation of cancer cells in vivo [71].

In contrast, Yan et al. fabricated ICG-loaded GO for the application of PA-based tumor therapy [74]. The absorption spectrum of GO–ICG showed 10-fold enhanced absorbance compared to pristine GO at its peak region (780 nm) because of ICG’s FL quenching by the resonance energy transfer of GO. Therefore, GO–ICG efficiently converted the irradiated NIR laser into an acoustic wave and produced a 5-fold stronger PA wave than GO alone in water. Moreover, the integrin αvβ3 monoclonal antibodies were conjugated to the GO–ICG composites to enable selective tumor cell targeting by apoptosis-mediated cancer ablation. Meanwhile, chitosan (CS) is a natural polymer utilized in wound healing, antimicrobial activities, tissue engineering, and drug delivery systems owing to its superior biocompatibility and biodegradability [75,76,77,78].

Jun et al. developed FA-conjugated and CS-functionalized GO nanocomposites (FA–CS–GO) for PA-guided PTT application [79]. The FA and CS were conjugated by the EDC/NHS method and physically adsorbed to GO by strong hydrogen bonding and electrostatic interaction between the hydroxyl groups and amino groups of CS and negative charges on the surface of GO [80]. Subsequently, FA was conjugated to CS via a covalent bond using carbodiimide chemistry for targeting folate receptors. The fabricated FA–CS–GO showed enhanced stability in water and excellent biodistribution in MDA-MB 231 tumor-bearing BALB/c nude mice. After 20 days of intravenous injection (i.v.) of FA–CS–GO, the H&E staining of the heart, spleen, liver, kidney, and lung indicated there were no obvious organ damage and recurrence of tumors. The biodistribution of G-based nanocomposites was also evaluated by Jin et al. and Zhang et al., suggesting the safety of FA–CS–GO for in vivo application [81,82]. CT is one of the most frequently used medical imaging tools, owing to its deep tissue penetration with suitable resolution and the capability of three-dimensional (3D) images for cancer diagnosis [83]. Generally, CT requires the renal injection of a contrast agent composed of iodinated small molecules with a high X-ray absorption coefficient; however, renal toxicity and rapid clearance limit the application of iodine agents [84,85]. Bismuth is a biocompatible heavy metal that exhibits high X-ray attenuation and CT contrast efficacy. For the complementary imaging of an accurate diagnosis of cancer, PAI can be combined with conventional CT.

Zhang et al. introduced a nanotheranostic agent composed of GO, PVP, and bismuth selenide (BI2Se3) for versatile applications, including CT, PAI, and PTT [86]. The GO/Bi2Se3/PVP nanocomposites exhibited cytocompatibility at a concentration under 150 μg/mL to HeLa cells and showed little hemolytic activity and in vivo accumulation in HeLa tumor-bearing nude mice. The GO/Bi2Se3/PVP nanocomposites could serve as an efficient bimodal contrast agent to enhance X-ray CT imaging and PAI in vivo. At the same time, irreversible PT ablation of tumors in the mouse model was achieved by using 808 nm laser irradiation.

The other study conducted by Chang et al. enabled photothermo-chemotherapy by combining PTT, MRI, and PAI in a single probe composed of GO/MnWO4 [87]. Among the MRI contrast agents, T1 agents such as Gd(III)-based or Mn(II)-based agents are known to possess higher contrast than T2 agents due to fewer artifacts and higher signal intensity [88,89,90]. In this work, MnWO4 was grown on a GO surface in a hyperthermia polyol medium containing PEG (GO/MnWO4/PEG) to enhance biocompatibility and water distribution. The fabricated GO/MnWO4/PEG nanocomposites showed a high DOX loading capacity that features pH- and NIR-stimulated drug release with suitable clearance and water contents. The released DOX and PT activities of GO/MnWO4/PEG significantly ablated mouse tumors in vivo, suggesting the versatility of GO/MnWO4/PE for multimodal imaging-guided cancer synergistic therapy. On the other hand, AuNPs have tunable and enhanced adsorption cross-sections, making them preferred versatile imaging probes for MRI, CT, PA, and FL imaging [91,92,93,94]. However, a reduction in the cross-section of AuNPs hinders the acceptable level of PA signal amplitudes under the visible light range. While maintaining the overall compact size, GO can be conjugated with AuNPs to enhance adsorption cross-sections to augment PA signal generation.

Sreejith et al. reported GO-wrapped silica-coated AuNPs (GO–AuNP@SiO2) that exhibit absorption enhancement in the visible region of the electromagnetic spectrum [95]. Upon increasing the size of the AuNPs at the core (10 to 30 nm), the PA signal of GO–AuNP@SiO2 was noticeably increased (2.3-fold) compared to AuNP@SiO2. Furthermore, the bimodal imaging of PA and FI modes revealed an enhanced PA signal from GO–AuNP@SiO2 embedded in gelatin-separated wells in agar phantoms. The results indicate that the photoluminescence, PA signal, and electromagnetic field can be sophisticatedly tailored by changing the size of the particles. Meanwhile, a different formulation of GO and silica nanocomposites was developed by Nguyen et al. for the versatile application of PAI and TPI [96]. TPI offers excellent optical sectioning with minimal cellular photodamage for highly sensitive and long-time cell imaging [97]. The mesoporous silica was grown on both sides of the GO to fabricate a nano-sandwich; TPI-active dye 1 (4-(4-diethylaminostyryl)-1-methylpyridinium iodide) was loaded onto the mesopores and coated with PAA (PAA@NS1). The PAA coating was applied for the prevention of dye leakage and better aqueous compatibility. The unique properties of PAA@NS1 composites of loaded TPI-active dye and NIR absorption enabled the dual-modal imaging of TPP and PAI in chicken tissue models.

Compared to GO, the most advantageous feature of rGO is high loading efficiency due to its rich π–π conjugation structures, which enable the effective adsorption of aromatic molecules such as DOX and ICG by π–π stacking and hydrophobic interactions [98,99,100,101]. Moreover, strong optical absorption in the NIR region makes rGO-based composites promising PAI probes [102].

**Figure 5:**
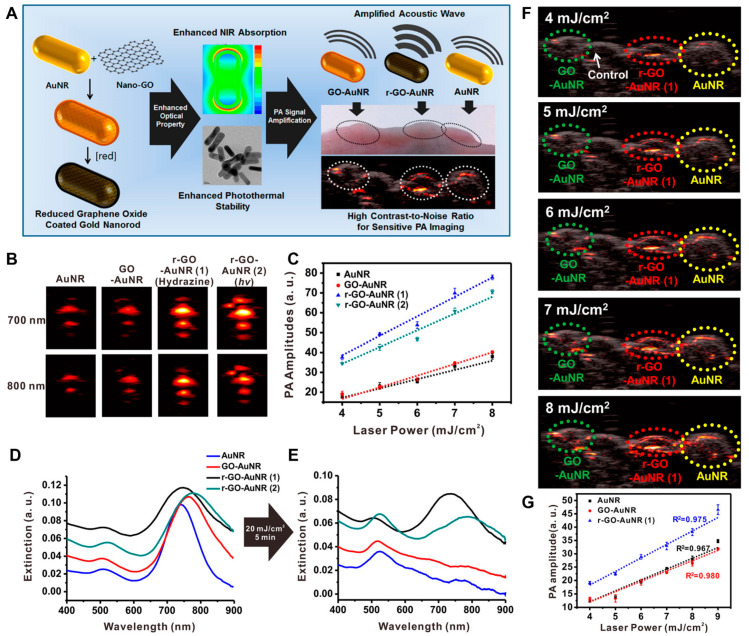
rGO–AuNRs for PAI application. (**A**) Synthesis and characterization of rGO–AuNRs and in vivo mouse PAI. (**B**) Representative PA images obtained at 700 and 800 nm laser irradiation and (**C**) corresponding PA amplitude. UV–visible spectra (**D**) before and (**E**) after laser illumination for 5 min (20 mJ/cm^2^, 10 Hz). (**F**) PA images visualized after illumination with different input laser powers that ranged from 4 to 8 mJ/cm^2^ in living mice. (**G**) Quantitative analysis of photoacoustic signal intensities obtained with each group. The images are reproduced with permission from ref. [103].

Moon et al. investigated the potential of rGO-coated AuNRs (rGO–AuNRs) for sensitive PAI and PTT applications (Figure 5) [103]. Owing to the excellent NIR light adsorption properties of rGO–AuNRs, PA signal intensity increased up to 1.5-fold compared to bare AuNR at the mouse intradermal. Theological studies revealed that a 4-times higher magnitude of electromagnetic field was generated compared to bare AuNRs or GO–AuNRs. High PA amplitudes were observed in the 4–11 MHz range, which is the operating frequency of the ultrasound transducer, suggesting the fabricated rGO–AuNRs can be a multimodal deep-tissue imaging probe.

In the other study conducted by Hu et al., ICG-loaded PDA–rGO nanocomposites (ICG–PDA–rGO) were fabricated, and the PA and PT effects were noticeably amplified [102]. Generally, GO and rGO have a broad absorption spectrum that leads to low PA resolution and PT conversion efficiency. To overcome the issues, ICG-conjugation can be exploited to enhance the light absorption efficiency of rGO at the plasmon frequency to amplify PA and PT performances. Dopamine is a natural reducing agent that helps to reduce the process of GO through a self-polymerization reaction and makes a PDA layer coating on the surface of rGO [104]. The resulting ICG–PDA–rGO showed a 20-fold increased PA signal compared to bare GO at mouse tumor sites. The 4T1-tumors in the subcutaneous and orthopedic mice models were ablated by PAI-guided PTT treatments. During the process, no toxicity was observed.

**Figure 6:**
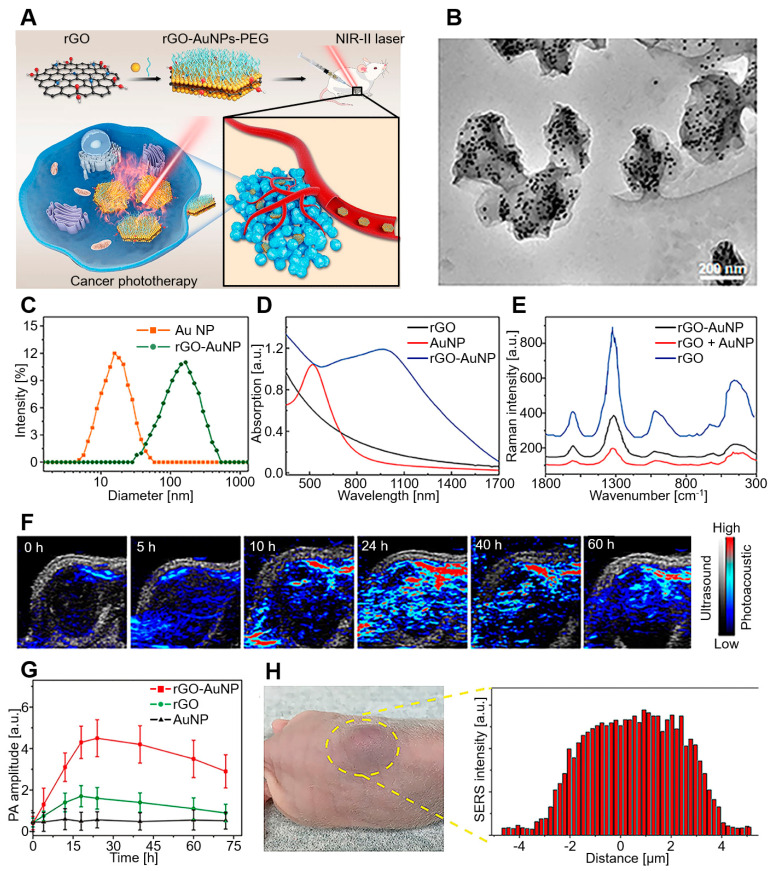
rGO–AuNP–PEG for PAI in the NIR-II window. (**A**) Schematic diagram of rGO–AuNP–PEG synthesis and PAI-guided PTT for cancer ablation in the NIR-II window. (**B**) TEM images, (**C**) hydrodynamic distribution, (**D**) UV–vis spectra, and (**E**) SERS spectra of rGO–AuNPs. (**F**) PA images and (**G**) PA amplitude after 1250 nm laser irradiation. (**H**) In vivo SERS signal of the tumor and its surrounding tissues to determine the boundary between tumor and normal tissue. The images are reproduced with permission from ref. [105].

Wang et al. developed a sandwich nanostructure of AuNP-coated rGO for PAI-guided PTT in the second NIR (NIR-II) window (Figure 6) [105]. NIR-II is a light wavelength of 1000 to 1700 nm that has been increasingly explored due to its potential for preclinical and clinical applications. Compared to the visible (400–700 nm) and NIR-I (700–1000 nm) areas, the NIR-II window offers several advantages, including high spatial resolution, deep tissue penetration capacity, and decreased optical absorption or scattering by tissues [106,107,108,109]. The rGO–AuNP nanocomposites exhibited greatly increased PA and PT effects in the NIR-II window due to the strong plasmonic coupling of AuNPs and electromagnetic fields generated by rGO. Furthermore, dissociation of rGO and AuNPs after laser irradiation enabled fast clearance after cancer theranostics. On the other hand, gene delivery is an advantageous therapy in terms of treatment for the root of diseases by correcting or compensating for abnormal genes [110,111]. For the successful delivery of genes, the development of a vector that can escort genes safely to cells is essential [112,113,114].

Jia et al. synthesized G@AuNS/lipid for the delivery of the pancreatic cancer gene; it features synergistic therapy with PA/PT imaging dual-modal guidance [115]. The K-Ras gene mutation is a commonly found mutation in pancreatic cancer that plays an important role in the growth and proliferation of cancer cells [116,117]. To target the K-Ras mutated cells, FA-functionalized lipid (FA-DODAB/DOPE) was coated onto G@AuNS (rGADA) to deliver the G12V (glycine to valine mutation of the K-Ras oncogene on codon 12) mutant anticancer K-Ras gene plasmid (KrasI) for the pancreatic cancer gene. The results indicated that the crosslinking of FA on the rGADA surface could specifically bind to the FA rectors of cancer cells and induce receptor-mediated endocytosis. Subsequently, PAI-guided synergistic treatment of the G12V mutant K-Ras gene and PTT showed excellent cancer ablation efficiency for pancreatic cancer tumor-bearing mice. During the process, rGADA did not induce necrosis and apoptosis in the surrounding cells, indicating the biocompatibility of particles.

The multimodal theragnosis probe can be achieved by the combination of rGO and AuNRs. Song et al. fabricated rGO-loaded plasmonic AuNRVe with a high capacity loading of DOX (rGO–AuNRVe/DOX) for cancer theragnosis [104]. The loaded DOX–rGO conjugate is released from the vesicular cavity by NIR-induced PT heating, and the intracellular acidic environment induces DOX release from the surface. Moreover, the versatile characteristics of rGO–AuNRVe/DOX enable multimodal imaging systems, including FL, US, and PET. Notably, rGO–AuNRVe/DOX showed a 20-fold increased PA signal compared to AuNRs and exhibited suitable cytocompatibility at 6.4 μg/mL to U87 MG cells. The results suggest that rGO–AuNRVe/DOX is capable of clinical translation to treat cancer patients with tumors accessible by light.

The other study conducted by Lin et al. also focused on PAI-guided PTT by combining PEGylated rGO and GSP (PEG–rGO–GSP) [118]. GO was used as an emulsifying agent for the fabrication of GSP with the NIR method and spontaneously deposited on the surface of GSP as a precursor of the rGO shell. The plasmonic coupling of rGO–GSP enhanced PT conversion properties that led to sensitive PA detection and excellent PT ablation of U87MG tumor-bearing BALB/c nude mice in vivo. At this time, PEG–rGO–GSP showed a 10-fold increased PA signal compared to control, and the cytotoxicity was evaluated by live/dead assay, indicating 100 μg/mL is not cytotoxic. Consequently, colloidal superparticle–G hybrid nanostructures can also be a promising probe for PAI-guided cancer therapy.

In contrast, Sheng et al. focused on the fabrication methods of rGO imaging probes using BSA [119]. BSA is a natural active protein that can be utilized as a reductant and stabilizer for the synthesis of hybrid nanoclusters [120]. Due to exceptional advantages such as low cost, availability, high solubility in water, and good ligand-binding property, BSA can be a reduction agent for the fabrication of rGO [121]. The fabricated nano-rGO showed PA/US dual-modality imaging feasibility and has been utilized as a PAI-guided PT agent and as an i.v.-administered theranostic probe in tumor-bearing mice. Compared to blood, a 1.5-fold enhanced PA signal was observed at the mouse tumor site by passive targeting. Moreover, NIR irradiation induced PT effects on cancer cells, while there was no noticeable cytotoxicity to cells under a concentration of 80 mg/L. Meanwhile, the superparamagnetic property refers to magnetic moments in the presence of an external magnetic field; otherwise, the net magnetic moment is zero [122]. For MRI application, superparamagnetic IONPs were highlighted due to their biocompatibility and strong effects on T2 and T2* relaxation, allowing sensitive and accurate tracking of the labeled cells [123,124,125].

Yang et al. developed an rGO–IONP complex that is noncovalently functionalized with PEG (rGO–IONP–PEG) [126]. In addition to excellent physiological stability and strong NIR optical absorbance, the superparamagnetic properties derived from the IONP complex enabled its multimodal application as an MRI probe. The in vivo triple modal, FL, PA, and MRI, was conducted with passive tumor targeting; this resulted in the effective photothermal ablation of 4T1-tumors in mice.

GQD is a kind of quasi-zero-dimensional CNM derived from G that possesses exceptional optical properties [127]; its special edge effects and surface structure endow it with many physicochemical properties, including rich photoinduced electron transfer, oxidation-reduction performance, and FL emission properties [128,129]. Compared to other kinds of QDs, GQD has superior biocompatibility, FL stability, and tailored chemical and surface properties, which make it suitable for bioimaging applications [130,131,132,133]. Xuan et al. developed FA-coupled N-doped GQD (N–GQD–FA) that featured high PT conversion efficiency for dual-modal imaging [127]. The fabricated N–GQD–FA showed uniform and small-sized (5 nm) morphology, and a concentration of under 500 μg/mL was proven to be cytocompatible with HeLa and A549 cells. Notably, N–GQD–FA shows excellent PT conversion efficiency and high FL quantum yield in the NIR region, which means FL-PA dual-modal in vivo imaging is simultaneously capable of being applied in NIR light irradiation. At the same time, the coupled FA allows the passive targeting of cancer cells, suggesting the great application prospects of cancer theranostics.

In summary, G-derivatives, including GO, rGO, and GQD, have been utilized for PAI-guided imaging and therapy. According to the fabrication method and modification, GO- and rGO-based probes have shown 1.5- to 20-fold PA intensity and multimodal potential as FL, PTT, CT, MRI, chemotherapy, TPI, PET, US, and SERS probes. Notably, PVP, PEG, and BSA-functionalized probes have shown enhanced cytocompatibility (up to 80–200 μg/mL), while GQD probes need to be further investigated. The characteristics of G-derivatives are summarized in Table 2.

## 7. Carbon-Nanotube-Based Photoacoustic Imaging

The CNT consists of a single layer of rolled-up G, such as in cylinder-like morphology. With the recent discovery of the versatile properties of CNTs, they have revolutionized biomedical research with their impressive structural, mechanical, and electrical properties [134,135,136,137,138,139]. Notably, their high drug-loading capacity due to their large surface-volume ratios, as well as their high mechanical properties and electrical and thermal conductivities, show their potential in PAI. The low solubility of CNTs is their main hurdle, and several research methodologies have focused on the conjugation of bioactive molecules to suggest novel manipulations to solve the issue [140,141].

**Figure 7:**
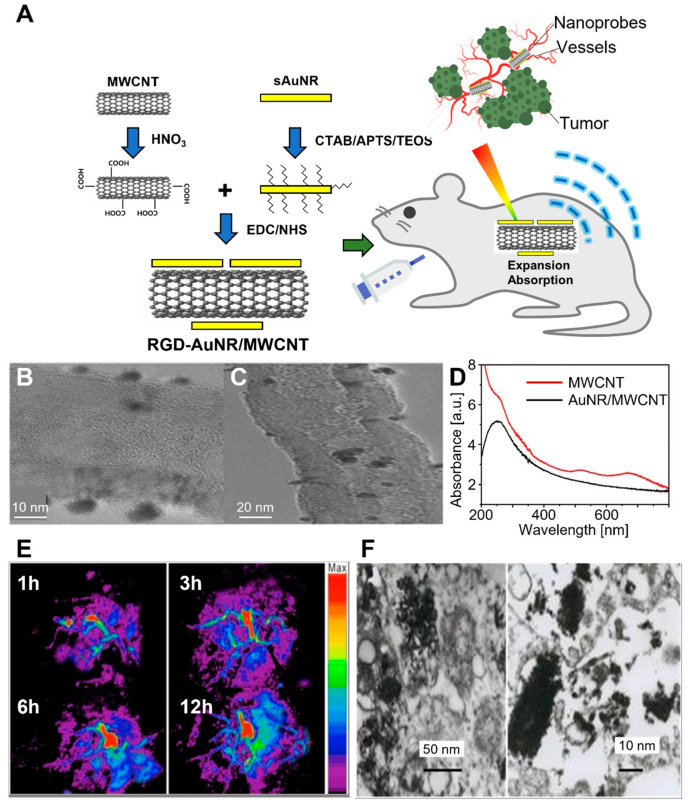
RGD–AuNR/MWCNT conjugate for targeted PAI of gastric cancer. (**A**) Schematic diagram of synthesis and application of RGD–AuNR/MWCNT in mouse gastric cancer vessel visualization. (**B**) TEM and (**C**) HR–TEM images of AuNR/MWCNT. (**D**) UV–vis spectra of pristine MWCNT and AuNR/MWCNT. The inset shows the magnification in the region of 400~800 nm. (**E**) Photoacoustic images of different post-injection times. (**F**) TEM images of RGD–AuNR/MWCNT inside the MGC803 cells. The images are reproduced with permission from ref. [142].

Wang et al. developed RGD-conjugated silica-coated AuNRs on the surface of MWCNT (RGD–AuNR/MWCNT) for PAI-guided gastric cancer therapy (Figure 7) [142]. The composites were fabricated via crosslinking between the carboxyl groups of MWCNT and the amino group of AuNRs, with subsequent modifications with silane coupling agents and RGD peptides. The in vitro cytotoxicity assay indicated that 200 μg/mL of RGD–AuNR/MWCNT was cytocompatible for MGC803 and GES-1 cells. Subsequently, RGD–AuNR/MWCNT was injected into the tail vein, and the MGC803 tumor-bearing mice were observed by an optoacoustic imaging system. The RGD–AuNR/MWCNT exhibited enhanced water solubility and cancer cell targetability, with strong PA signals and PT properties in vivo. On the other hand, SLN biopsy is a less invasive alternative for the treatment of clinically node-negative breast cancer [143].

Generally, lymphatic mapping with radio-labeled sulfur colloid or blue dye is widely used; however, its reliance on invasive surgical procedures with associated morbidity is still a problem. To solve the issue, Pramanik et al. developed the potential of SWCNT as a PAI probe for SLN visualization [144]. The injected SWCNT in Sprague–Dawley rats exhibited a 4-fold increased PA signal compared to blood at the excitation wavelength range of 740–820 nm. Moreover, varying the wavelength of incident light at the NIR region helped low light absorption into the surrounding biological tissue while the imaging depth was maximized.

The other study conducted by Cai et al. evaluated the potential of SWCNT for multiscale PAI and its capability as a tissue engineering scaffold [145]. Both acoustic-resolution PAI and optical-resolution PAI were employed to image the SWCNT/PLGA scaffolds immersed in biological buffer. At the excitation wavelength of 570–638 nm, the multiscale PAI excellently differentiated PA signals generated from blood and CWNCT/PLGA scaffolds. The results suggest that PAI is a promising non-invasive tool and a real-time imaging tool for tissue engineering scaffolds in vitro as well as in vivo under physiological conditions.

Zamganeh et al. evaluated the efficiency of PAI-guided delivery of ICG to the tumor site, conjugated to SWCNT (ICG/SWCNT) [146]. It was shown that ICG provided 33% enhancement at an approximately 20 min peak response time, while ICG/SWCNT provided an enhancement of 196% at 120 min on average. The PA signal of ICG/SWCNT was 2-fold increased compared to bare ICG, mainly at the tumor periphery, suggesting that it could provide guidance to surgeons when assessing tumor boundaries.

Meanwhile, to endow the PA probe with cancer targetability, targeting αvβ3 integrins that are associated with tumor angiogenesis could be a brilliant method. Zerda et al. introduced SWCNT–ICG–RGD conjugates as tumor-targetable PA contrast agents [147]. The SWCNT–ICG–RGD composites provided a 300-fold increased PA signal in tumor-bearing mice compared to bare SWCNT, indicating that their αvβ3-integrin-mediated cancer targeting is highly efficient.

The other study conducted by Zerda et al. fabricated RGD-coupled PL–PEG5000-conjugated SWCNT probes (SWCNT/RGD/PL–PEG5000) for PAI imaging [148]. Due to the presence of RGD peptides, the conjugated probes exhibited a high affinity to the αvβ3 integrin, which is overexpressed in the tumor neovasculature process [149,150]. The i.v.-injected SWCNT/RGD/PL–PEG5000 showed an 8-fold increased PA signal in U87MG tumor-bearing mice compared to bare SWCNT. Notably, the advantages of the PA strategy were compared with the FL imaging of tumor-targeted quantum dots (RGD-QDs). Six hours after the injection, the PA images illustrated greater depth information and spatial resolution compared to FL images because they could avoid the smeared signal derived from light scattering.

Song et al. reported CNTR-coated AuNPs that use CNTR as the template for the attachment of redox-active polymers as a reducing agent and subsequent metallization by AuNP coating (CNTR@AuNPs) [151]. Due to their hierarchical structure, the CNTR@AuNPs could be applied as highly enhanced and versatile imaging probes for optical imaging, Raman spectroscopy, and PAI. The extinction intensity of CNTR@AuNPs at 808 nm was 120-fold higher than bare CNTR, and the SERS signal of CNTR@AuNPs was about 110-fold increased compared to CNTR, suggesting the synergistic coupling of the embedded CNTR and the plasmon mode of the closely attached AuNPs.

Zanganeh et al. focused on the hypoxia targeting of SWCNT as a sensitive contrast agent for the PAI of tumors; 2nitroimidazole–ICG was conjugated to SWCNT (SWCNT–2nitroimidazole–ICG), a unique hypoxic marker that is an important indicator of tumor metabolism and tumor therapeutic responses [152,153,154]. Forty minutes after injection, SWCNT-2nitroimidazole-ICG exhibited a 6-fold enhanced PA signal; 100 min after injection, the signals were 7.7-fold increased; at 250 min after injection, the signals were still 2.5 times stronger than at the time of injection. The results indicate that the dye-conjugation of the PA probe not only increases the PA signal but also helps it stay longer through the hypoxic area of the tumor.

Nozdriukhin et al. developed a AuNP–CNT multilayer on silica microspheres for a PA-Raman enhanced probe [155]. The PA-Raman bimodal contrast agents were fabricated by the layer-by-later self-assembly method of CNT and AuNPs around the silica–PDDA/PSS/PDDA microsphere ((PDDA/PSS/PDDA–silica)/CNT/AuNP). The sandwiched CNT between AuNPs exhibited strong light absorption in the visible and NIR regions, with high PA contrast at 532 nm and Raman scattering at 785 nm, respectively. Moreover, the PA flow cytometry of (PDDA/PSS/PDDA–silica)/CNT/AuNP significantly increased under the laser wavelength of 1064 nm, and subsequently, the results were validated by ex vivo brain tissue using a portable Raman spectrometer and imaging with Raster-scanning PA mesoscopy. These results suggested that the fabricated (PDDA/PSS/PDDA–silica)/CNT/AuNP can be a promising contrast agent for PA tomography, PA flow cytometry, and multiplex SERS detection.

Because most CNT-based probes feature low water solubility and relatively high toxicity compared to other CNMs, many studies use functionalization strategies with biocompatible polymers such as PLGA and PEG or RGD peptides. Due to their high drug/dye loading capacity, ICG, 2-nitroimidazole, and AuNPs were conjugated to enable PAI-guided therapy, such as FL, CT, and Raman spectroscopy. Despite their high PA signal-reinforcing property (up to 30-fold), their cytocompatibility and in vivo toxicity should be further elucidated. The characteristics of the CNT-based materials are summarized in Table 3.

## 8. Carbon-Nanoparticle-Based Photoacoustic Imaging

CDs or carbon nanodots (CNDs) have been widely applied as bioimaging probes owing to several desirable properties, such as excitation wavelength-dependent luminescence emission, low photobleaching, low toxicity, suitable in vivo clearance, high stability, and solubility [156,157,158,159]. However, the main absorption band of CNDs is UV to the green region, and it is important to develop methods to endow CNDs with high absorption coefficients in the red to NIR region for deep tissue penetration for PTT and PA imaging [160,161,162].

**Figure 8:**
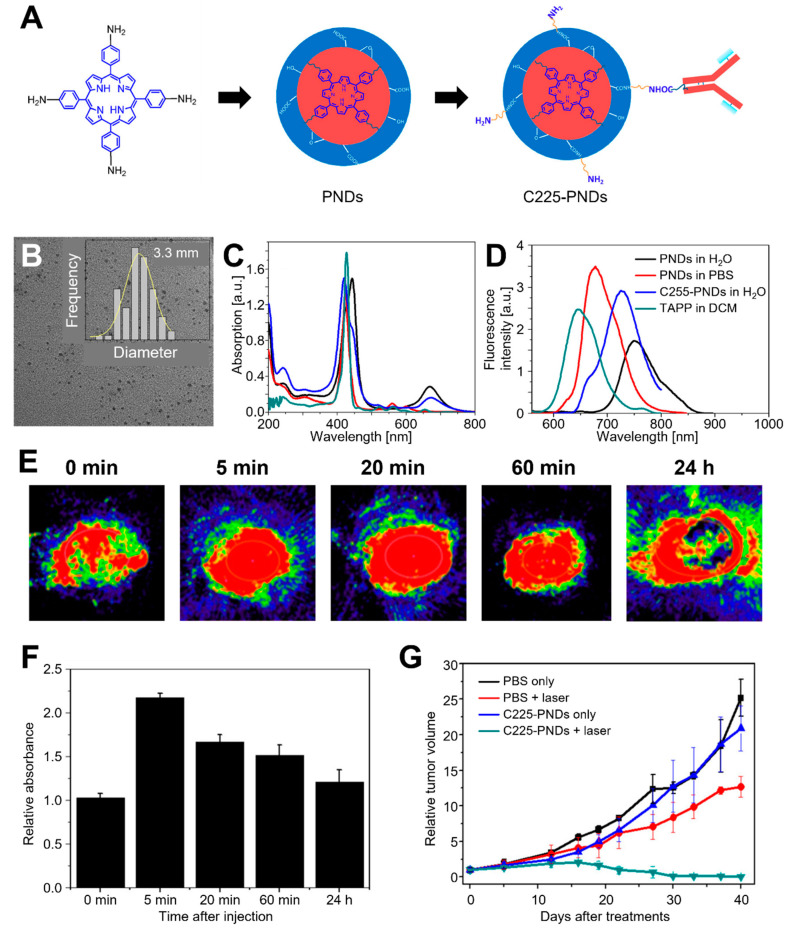
C225–PNDs for PAI and in vivo breast cancer ablation. (**A**) Proposed formation pathway of PNDs and the synthetic routes of C225–PNDs. (**B**) TEM image of PNDs with a corresponding size distribution histogram. (**C**) UV–vis spectra and (**D**) emission spectra (λex = 440 nm) of PNDs, C225–PNDs, and 5,10,15,20-tetrakis (4-aminophenyl) porphyrin (TAPP). (**E**) PA imaging and (**F**) relative PA intensity of C225–PNDs in the tumor at different time points. (**G**) Relative tumor volume. The images are reproduced with permission from ref. [165].

To magnify the PA signal of CD-based probes, many porphyrin photosensitizers are utilized [163,164]. However, many of them are limited in clinical application due to prolonged cutaneous photosensitivity, poor water solubility, and low selectivity and fluorescence quantum yield. To overcome these issues, Wu et al. developed porphyrin- and cetuximab-containing CNPs (C225–PNDs) through the supramolecular self-assembly of lipid- or peptide–porphyrin conjugates (Figure 8) [165]. The fabricated C225–PNDs showed excellent stability and biocompatibility with the characteristic properties of porphyrin, which are strong UV–visible and NIR light absorption. The C225–PNDs could actively target EGFR-overexpressed cancer cells, leading to highly efficient PDT upon two-photon excitation at 800 nm laser irradiation. Moreover, PAI-guided PDT on MDA-MB-231 breast tumor-bearing mice showed complete tumor ablation, indicating the clinical potential of C225–PNDs.

Hua et al. investigated the multimodal properties of a novel type of CD, which was synthesized by mixing BSA, CD, and metal ions, including Cu2+ and Gd3+, followed by conjugation with an HPPH (BCCGH) [166]. The BCCGHs showed excellent multimodal potential for FL, PA, MR, and PT imaging-guided PTT/PDT synergistic therapy. During in vivo imaging, BCCGHs showed high PT conversion efficiency (68.4%), longitudinal relaxivity (11.84 mM^−1^ s^−1^, 7 T), and superior colloidal stability. Moreover, due to the facilitation of cellular uptake, BCCGHs are easily delivered to the nucleus because of their NIR-medicated endoplasmic reticulum/mitochondrion-targeting properties. Both in vivo and ex vivo experiments demonstrated that BCCGHs have excellent accumulation, targeting, and ablation characteristics with biocompatibility and suitable renal clearance, promising great potential for cancer theranostics. Meanwhile, non-metallic elements such as N, S, P, and Si can be doped into CDs to enhance their optical properties [167,168,169].

However, metal dopants doped to CDs are still limited despite their optical advantages. Yang et al. developed Mn-doped nigrosine-originated CDs (Mn–NCDs) for FL and PA dual-modal imaging-guided PTT application [170]. The fabricated Mn–NCD had an average particle size of 3 nm and a long emission wavelength of 653 nm. The MN–NCD exhibited suitable biocompatibility, with a low hemolysis level and cytocompatibility under a concentration of 1 mg/mL. Notably, when the external NIR lights were irradiated, the temperature of the MN–NCDs increased from 23.1 to 50 °C, indicating they can generate enough hyperthermia to efficiently ablate tumor cells both in vitro and in vivo.

Jia et al. fabricated AuNR@SiO2–CDs as a PA agent by incorporating CDs with AuNRs using SiO2 as a scaffold [171]. The AuNR@SiO2–CDs exhibited non-cytotoxicity at a concentration under 100 μg/mL toward B16-F0 cells, which was assessed by MTT assay and live/dead assay. Noticeably, the combination with AuNRs helped the particle act as not only PAI and PTT agents but also FL imaging and PDT agents. Moreover, the introduction of SiO2 greatly enhanced the chemical stability of the probe in the physiological environment and prevented the FL quenching of CDs by AuNRs. The i.v.-injected AuNR@SiO2-CDs in B16-F0 tumor-bearing nude mice exhibited high sensitivity and potential for high-resolution FL/PAI-guided PDT/PTT treatment, which promises the potential of AuNR@SiO2-CDs as novel phototheranostic agents for multifunctional cancer therapies.

CNSs have several advantages in being employed as PAI probes compared to G nanosheets, CNTs, and other kinds of CNMs [172,173,174]. CNSs are intrinsically biocompatible due to their hydrophilic surface; there is no need for additional surface treatments. Moreover, the sphere shapes of CNSs are beneficial for the treatment of solid tumors with a homogeneous existence in the margin.

Miao et al. demonstrated the fabrication of glucose-derived CNSs for PAI-guided PTT [175]. The hydrothermal treatment of glucose enables the synthesis of colloidal CNSs with a tunable diameter. Because of the use of glucose as a carbon source and water as a solvent, the synthesized CNSs were highly biocompatible and nontoxic under 320 μg/mL. The injection of CNS into 4T1 tumor-bearing mice showed a 2.5-fold increased PA signal compared to preinjection. These results suggest that CNSs are suitable for PAI-guided PT ablation of cancer cells and can be served as promising biocompatible carbon-based agents for further clinical trials.

Wu et al. also focused on the green synthesis of CNPs for real-time PAI of SLN [176]. The CNPs were obtained from commercial food-grade honey for the first time. To fabricate the luminescent CNPs with rapid clearance properties, the one-pot green technique was used, which involved the rapid surface passivation of CNPs with organic macromolecules, including polysorbate and PEG (OCN), in solvent-free conditions. The resulting OCNs were markedly smaller (~7 nm) than previously reported particles, such as gold, copper, and CNTs, for SLN imaging [177,178,179]. Owing to their tiny sizes, OCNs showed rapid lymphatic transport and clearance, with strong optical absorption in the NIR region. The fast and high-resolution imaging with OCNs shows great potential for the faster resection of SLN and lower complications caused in axillary investigations by mismarking with dyes or low-resolution imaging techniques.

NDs have an sp3-carbon diamond core and a reconstructed sp2-carbon surface layer with a diameter of 4–5 nm, featuring high energy absorption properties [180]. The reconstructed sp2-carbon surface serves as a crystalline carbon with hybrid orbitals, and the dangling surface functional groups exhibit extraordinary activities, providing a large area to exploit multiple functions [181,182]. Lee et al. developed AuNP-conjugated NDs for enhanced PA and Raman dual-modal imaging (NDAuNPs). MTT assays indicate that NDAuNPs are cytocompatible under a concentration of 125 μg/mL to C2C12 and A549 cells, with excellent biodegradation properties. The fabricated NDAuNPs showed a 1.5-fold increased PA signal in chicken breast muscles and SERS-enabled enhanced Raman intensity in a concentration-dependent manner. Notably, the PA signal of NDAuNPs was maintained during prolonged periods of laser irradiation and impeded the degradation of Au without PA signal decay.

Zhang et al. introduced PEGylated and HER2-functionalized NDs (HER2–PEG–NDs) as active tumor targeting-enhanced PAI probes. Because of the specific binding of NDs to HER2-overexpressing 4T1.2 neu murine breast cancer cells, tumor tissues are significantly visualized from the surrounding normal tissue, which exhibited a 1.3-fold increased PA signal compared to bare PEG–ND at a wavelength of 820 nm. Moreover, a longer retention time of HER–PEG–NDs was observed in the HER2-overexpressing tumor model than in the negative tumor model (4T1.2), suggesting that HER2 targetability has a great potential for sensitivity in the in vivo detection of tumors.

Cui et al. developed multicomponent HA, PS, NDs, Cur, and IR780 into a single nanoplatform (denoted as HPNDIC) based on the combination of hydrophobic and electrostatic noncovalent interactions for the FL/PAI dual-modal imaging-guided chemical/PTT/PDT combination therapy of triple-negative breast cancer (TNBC) [183]. The fabricated HPNDICs showed uniform size, high drug-loading ability, and excellent colloidal stability. The in vitro and in vivo biocompatibility assays indicated they were cytocompatible under 100 μg/mL, and there was no significant hemolysis, organ accumulation, and damage in mice, respectively. Investigations confirmed that NIR could induce IR780-triggered PTT/PDT dual therapeutic effects combined with Cur delivery, while the biodistribution and accumulation behavior of HPNDIC in vivo could be monitored by dual-modal FL/PAI. These results indicate that HPNDICs could be a novel multicomponent theranostic platform for dual-modal imaging-guided triple-collaborative therapy for TNBC treatments.

In summary, C-based NPs comprise many forms, such as CDs, CNDs, CNSs, CNPs, and NDs. Due to their spherical morphology, water stability and biocompatibility (100–1000 μg/mL) were shown to be superior to other types of CNMs. The FL property of CDs, CNDs, and NDs promises their cell imaging application. Proteins, drugs, and metals can be conjugated to enable FL, PDT, and MRI multimodal applications. Notably, NDs have natural nitrogen-vacancy (N-V) centers that induce complex formations in the core with peptides and amines for drug loading, which make them promising therapeutic probes. The characteristics of C-based NPs are summarized in Table 4.

## 9. Conclusions

PAI has attracted attention for its ability to investigate in vivo phenomena with improved intermediate properties of optical and US imaging. Pure endogenous chromophores allow excellent resolution in the visible and near-infrared window, but the rapid attenuation of the wavelength results in shallow penetration into biological tissue. The synthesis of novel exogenous contrast agents that can increase the depth of penetration into biological tissues while ensuring strong light absorption in various wavelength bands holds additional potential in PAI for clinical research. In this review, we discussed the cases of applying CNM-based nanocomposites such as G, CNTs, and CNPs in PAI. CNMs have excellent physical and electrochemical properties and enable the synthesis and loading of various functional materials and drugs, so they are widely being adopted as suitable PAI probes in vivo. G derivatives feature the highest surface-volume ratio and oxygen-containing functional moieties that enable efficient drug loading and biofunctionality. Meanwhile, CNTs interact with outer electromagnetic radiation in a unique way, which endows them with photoluminescence and Raman properties. Different types of CNPs can be utilized, such as CNDs, CNSs, and NDs, which can diversify their applications regarding imaging targets, biofunctionality, and physicochemical properties.

The recently developed CNM-based nanocomposites effectively absorb light in UV, visible light, and NIR-I, II windows. This extends the image resolution and penetration depth of PAI to a wider selectable range. In addition, multispectral analysis can also be performed in a more versatile range. Many cases have shown that in vivo monitoring, due to the emphasized PA intensity, can perform well in areas of interest such as drug delivery and tumor cell targeting.

A comprehensive understanding of biocompatibility and biodegradable material properties is required for the full clinical application of CNM-based PAI. Complex chemical processing and analysis are also important to control photostability and avoid in vivo accumulation for accurate and safe diagnoses. Notable studies that carefully consider these factors are ongoing. We believe that advances in CNM-based contrast agents for PAI will become more promising, helping to confirm clinical diagnoses and therapy designs.

## Figures and Tables

**Table 1:** Performance benchmarks of representative results of OR-PAM, AR-PAM, and PACT. OR-PAM, optical-resolution photoacoustic microscopy; AR-PAM, acoustic-resolution photoacoustic microscopy; PACT, photoacoustic computed tomography; N/A, not available.

Type	Lateral Resolution (μm)	Axial Resolution (μm)	Center Frequency (MHz)	Imaging Depth (mm)	Application	Ref.
OR-PAM	3.5	27	50	1.5	Ear (mouse)	[27]
	3	15	50	0.7	Brain (mouse)	[28]
	5	15	100	0.7	Ear (mouse)	[29]
	6	37.7	50	N/A	Ear (mouse)	[30]
AR-PAM	50	25	50	2.4	Tumor (porcine stomach)	[31]
	53	18	75	1.8	Ear (mouse)	[32]
	130	57	30	11	Internal organs (rat)	[33]
	590	150	5	25	Internal organs (mouse)	[34]
PACT	129	1490	2.25	N/A	Brain (mouse)	[35]
	525	124	21	27	Femoral nerve (mouse)	[36]
	1000	400	50	50	Tumor (mouse hindlimb)	[37]
	1200	205	3–12	30	Internal organs (rat)	[38]

**Table 2:** G-based PAI. Each research was classified by G types, modification, abbreviation, theragnosis multimodalities, PA signal enhancement, novelties, maximum cytotoxicity concentration, and test species. AuNP, gold nanoparticle; APTES, 3-aminopropyltriethoxysilane; AuNS, gold nanostar; BSA, bovine serum albumin; CCK-8, cell counting kit 8; CS, chitosan; DODAB, dimethyldioctadecylammonium bromide; DOPE, 1,2-dioleoyl-sn-glycero-3-phosphoethanolamine; DOX, doxorubicin; FA, folic acid; FL, fluorescence; GNP, graphene nanoparticle; GQD, graphene quantum dot; GSP, gold superparticle; ICG, indocyanine green; IONP, iron oxide nanoparticle; MRI, magnetic resonance imaging; NR, nanorod; ND, nanodiamond; NIR, near infrared; O-MWGNR, oxydized graphene multi-walled nanorod; PAA, polyacrylic acid; PBS, phosphate-buffered saline; PDA, polydopamine; PEG, polyethylene glycol; PET, positron emission tomography; PT, photothermal; PTT, photothermal therapy; PVP, polyvinylpyrrolidone; US, ultrasound; SERS, surface-enhanced Raman scattering; rGO, reduced graphene oxide; TPI, two-photon imaging.

Types	Materials Modification	Multimodalities	PA Enhancement (Fold/Control)	Novelties	Cytocompatible Concentration	Test Species	Ref.
GO	ICG and FA	PTT	10/PBS at cell	High absorbance in the NIR region and cancer targetability	≤20 μg/mL	HeLa	[71]
	ICG and integrin α_v_β_3_	FL	5/GO at water	FL quenching via FL resonance energy transfer, selective tumor cell targetability, and apoptosis-mediated cancer ablation	≤21.5 μg/mL	U87-MG	[74]
	FA and CS	PTT	N/A	Stability in water and biodistribution and long-term observation of tumor recurrence inhibition	N/A	MDA-MB-231-BALB/c nude	[79]
	Bi_2_Se_3_ and PVP	CT, PTT	N/A	Little hemolytic activity and in vivo toxicity and hydrophilicity	≤150 μg/mL	HeLa-BALB/c nude	[86]
	MnWO_4_ and PEG	MRI, PTT, chemotherapy	N/A	High drug loading capacity, pH- and NIR-stimulated drug release, and biodistribution and water content	≤200 μg/mL	HUVEC, 4T1, and 4T1-athymic nude	[87]
	Silica and AuNP	FL	2.3/AuNP@SiO_2_ at water	Controllable photoluminescence, high-resolution PA signal, and size-dependent electromagnetic field intensity	N/A	N/A	[95]
	APTES, silica, and PAA	TPI	N/A	High dose of dye loading and TPI for high-resolution depth penetrating imaging	N/A	HeLa, chicken breast	[96]
rGO	AuNR	PTT	2.5/AuNR at PBS	Higher light absorption and electromagnetic field generation and simultaneous application by overlapping absorbance peak for PA and US application	N/A	BALB/c mice	[103]
	ICG and PDA	PTT	20/GO at mouse tumor	Enhanced light absorbance by incorporation of ICG and In vivo tumor suppression without toxicity	N/A	4T1 and 4T1-BALB/c	[102]
	DOX and AuNR	FL, US, PET, PTT, chemotherapy	20/AuNR	Sequential drug release system, high drug loading capacity, and exceptional versatility	≤6.4 μg/mL	U87 MG and U87 MG-mouse	[104]
	AuNP and PEG	SERS, PTT	Mouse ovarian cancers	Availability in the second NIR region by plasmonic coupling and fast clearance due to particle dissociation	N/A	SKOV-3 and SKOV-3-BALB/c nude	[105]
	DODAB/DOPE–FA and AuNS	PTT	Mouse pancreatic tumor	PAI-guided PT/gene synergistic therapy (G12V delivery) and receptor-mediated cancer targeting	N/A	Capan-1 and Capan-1-BALB/c nude	[115]
	GSP and PEG	PTT, US	10/PBS	Exceptional chemical properties by plasmonic coupling of the self-assembled composites and GO-GSP emulsion method	≤100 μg/mL	U87MG and U87MG-BALB/c nude	[118]
	BSA	US, PTT	1.5/blood at mouse tumor	High stability and low cytotoxicity and passive targeting	≤80 mg/L	MCF-7 and MCF-7-mouse	[119]
	IONP and PEG	FL, MRI	N/A	Strong NIR absorbance and superparamagnetic properties	N/A	4T1-mouse	[126]
GQD	Nitrogen and FA	FL, PTT	3/N–GQD at cells	Uniform and small size (5 nm), strong quantum yield, and low cytotoxicity	≤500 μg/mL	HeLa and A549	[127]

**Table 3:** CNT-based PAI. Each piece of research is classified by CNT types, modification, abbreviation, theragnosis multimodalities, PA signal enhancement, novelties, maximum cytotoxicity concentration, and test species. CNTR, carbon nanotube ring; LBL, layer-by-layer; CD, carbon dot; CND, carbon nanodot; CNS, carboneous nanosphere; Cur, curcumin; HA, hyaluronic acid; SLN, sentinel lymph node; PDDA, poly (diallyl dimethyl ammonium chloride); PLGA, poly(lactic-co-glycolic acid); PL-PEG5000, polyethylene glycol-5000 grafted phospholipid; PSS, poly (sodium 4-styrene sulfonate); RGD, cyclic Arg-Gly-Asp.

Types	Materials Modification	Multimodalities	PA Enhancement (Fold/Control)	Novelties	Cytocompatible Concentration	Test Species	Ref.
MWCNT	RGD peptides and silica/AuNR	PTT	N/A	Good water solubility, cell targetability, and in vivo gastric cancer cell imaging	≤200 μg/mL	MGC803, GES-1, MGC803-BALB/c nude	[142]
SWCNT	RGD peptides and silica/AuNR	PTT	N/A	Good water solubility, cell targetability, and in vivo gastric cancer cell imaging	≤200 μg/mL	MGC803, GES-1, MGC803-BALB/c nude	[142]
	N/A	N/A	4/blood	SLN visualization	N/A	Sprague–Dawley rat	[144]
	PLGA	Micro CT	N/A	Availability as tissue engineering scaffold multiscale PAI	N/A	N/A	[145]
	ICG	FL	2/ICG at mouse breast tumor	Detailed optical characterization of SWCNT-reinforced probes	N/A	4T1 Luc, 4T1 Luc-BALB/c	[146]
	ICG and RGD	N/A	300/SWCNT at mouse subcutaneous region	α_v_β_3_-integrin-mediated cancer targetability and enhanced optical absorbance signal duration	N/A	Nude	[147]
	RGD and PL-PEG5000	N/A	8/SWCNT at ex vivo mouse tumor	Tumor targetability, non-invasive imaging of tumor, and absorbance in lower NIR window	N/A	U87MG-BALB/c nude	[148]
	CNTR, redox-active polymer, and AuNP	Optical microscopy, Raman microscopy	6/CNTR at water	Signal enhancement via local electrical field by SERS	N/A	U87MG cell and U87MG-nude	[151]
	PDDA, PSS, AuNP, and silica microsphere	Raman microscopy	N/A	AuNP–CNT multilayer assemble by LBL technique, strong absorption in NIR and visible region, and ex vivo mouse brain imaging	N/A	Human fibroblast C57Bl/6	[155]

**Table 4:** CNP-based PAI. Each piece of research is classified by CNP types, modification, abbreviation, theragnosis multimodalities, PA signal enhancement, novelties, maximum cytotoxicity concentration, and test species. HER2, epidermal growth factor receptor 2; HPPA, 2-(1-hexyloxyethyl)-2-devinyl pyropheophorbide-α; PS, protamine sulfate.

Types	Materials Modification	Multimodalities	PA Enhancement (Fold/Control)	Novelties	Cytocompatible Concentration	Test Species	Ref.
CD	Porphyrin and Cetuximab	PDT	N/A	Water stability, strong UV–vis and NIR abruption, and deep tissue penetration with high spatial resolution	≤100 μg/mL	HCC827, H23, MDA-MB-231, HBL-100, MDA-MB-231-mouse	[165]
	BSA–Cu_2+_–Gd_3+_ complex and HPPH	FL, MRI, PTT, PDT	N/A	In vivo mouse toxicity evaluation and decreased intracellular ROS generation	≤200 μg/mL	A549-BALB/c nude	[166]
	Mn and NCD	FL, PTT	N/A	Long emission wavelength and low hemolysis	≤1000 μg/mL	4T1-BALB/c mice	[170]
CND	AuNR and silica	FL, PDT, PTT	N/A	Chemical stability in a physiological environment and prevent absolute quenching of the FL	≤100 μg/mL	B16-F0 and B16-F0-nude	[171]
	Nitrogen	PTT	2/AuNR at water	Photostability, biodegradability, and SLN visualization	N/A	BALB/c nude	[184]
CNS	glucose	PTT	2.5/preinjection at mouse tumor	First investigation of CNS as PAI probe and controllable size of fabricated CNS	≤320 μg/mL	PC-3M-IE8, 4T1 and 4T1-nude	[175]
CNP	Honey, polysolvate, and PEG	N/A	N/A	One-pot green synthesis, markedly small probe for SLN imaging, rapid clearance properties, and rapid signal enhancement	N/A	Nude	[176]
ND	HA, PS, Cur, and IR780	FL, PTT, PDT	In vivo mouse tumor	Uniform size, high drug-loading ability, excellent colloidal stability, decreased hemolysis, and in vivo mouse toxicity evaluation	≤100 μg/mL	MDA-MB-231 and MDA-MB-231-mouse	[183]

## Data Availability

No new data were created or analyzed in this study. Data sharing is not applicable to this article.
